# A New Lower Bound for Noisy Permutation Channels via Divergence Packing †

**DOI:** 10.3390/e27111101

**Published:** 2025-10-25

**Authors:** Lugaoze Feng, Guocheng Lv, Xunan Li, Ye Jin

**Affiliations:** 1 State Key Laboratory of Photonics and Communications, Peking University, Beijing 100871, China; lgzf@stu.pku.edu.cn (L.F.);; 2 National Computer Network Emergency Response Technical Team/Coordination Center of China, Beijing 100029, China; lixunan@cert.org.cn

**Keywords:** noisy permutation channel, finite blocklength, divergence packing, *ϵ*-packing

## Abstract

Noisy permutation channels are applied in modeling biological storage systems and communication networks. For noisy permutation channels with strictly positive and full-rank square matrices, new achievability bounds are given in this paper, which are tighter than existing bounds. To derive this bound, we use the ϵ-packing with Kullback–Leibler divergence as a distance and introduce a novel way to illustrate the overlapping relationship of error events. This new bound shows analytically that for such a matrix *W*, the logarithm of the achievable code size with a given block *n* and error probability ϵ is closely approximated by $\ell \log\left(\frac{\sqrt{n}}{-\Phi^{-1}\left(\epsilon /G\right)}\right)+\log V\left(W\right)$, where ℓ=rank(W)−1, G=2ℓ+12, and V(W) is a characteristic of the channel referred to as channel volume ratio. Our numerical results show that the new achievability bound significantly improves the lower bound of channel coding. Additionally, the Gaussian approximation can replace the complex computations of the new achievability bound over a wide range of relevant parameters.

## 1. Introduction

The *noisy permutation channel*, consisting of a discrete memoryless channel (DMC) and a uniform random permutation block, was introduced in [1] and is a point-to-point communication model that captures the out-of-order arrival of packets. Such channels can be used as models of communication networks and DNA storage systems, where the ordering of the codeword does not carry any information. Previously, several advances have been made to asymptotic bounds, including the binary channels in [2], the capacity of full-rank DMCs in [1], and the converse bounds based on divergence covering [3,4].

The code lengths of suitable codes in communication systems are in the order of thousands or hundreds, invalidating the asymptotic assumptions in classical information theory. We initiate the study of new channel coding bounds to extend the information-theoretic results for noisy permutation channels in finite blocklength analysis. Finite blocklength analysis and finer asymptotics are important branches of research in information theory. Interest in this topic has been growing since the seminal works of [5,6,7]. These works suggest that the channel coding rate in the finite blocklength regime is closely related to the *information density* [8], i.e., the stochastic measure of the input distribution and channel noise. The second-order approximation of conventional channels involves the variance of the information density, which has been shown to approximate the channel coding rate at short blocklengths.

The codeword positions are randomly permuted in noisy permutation channels. Conventional analysis techniques, specifically the dependence-testing (DT) bound and the random coding union (RCU) bound ([9] Theorems 17 and 18), become inapplicable. Since the messages are mapped to different probability distributions in noisy permutation channels [1], the only statistical information the receiver would use from the received codeword $Y^{n}$ is which marginal distribution $Y^{n}$ belongs to. Therefore, the finer asymptotic completely differs from that in conventional channels.

The main contributions of our work are the following:

1. We present a new nonasymptotic achievability bound for noisy permutation channels that have strictly positive square matrices *W* with full rank. The two main ingredients of our proof are the following: the ϵ-packing [10,11] with Kullback–Leibler (KL) divergence as a distance, and an analysis for the error events that decouple the union of error events from the message set. Additionally, this new bound is stronger than existing bounds ([1] Equation (36)).
2. We show that the finite blocklength achievable code size can be approximated by
  (1) $$\log M^{*}\left(n,\epsilon \right)\approx \ell \log\left(\frac{\sqrt{n}}{-\Phi^{-1}\left(\epsilon /G\right)}\right)+\log V\left(W\right),$$
  where ℓ=rank(W)−1, G=2ℓ+12, and V(W) is the *channel volume ratio*.
3. To complement these results and assist in understanding them, we particularize all these results to typical DMCs, i.e., BSC and BEC permutation channels. Additionally, our Gaussian approximations, through numerical results, lead to tight approximations of the achievable code size for blocklengths *n* as short as 100 in these cases.

We continue this section with the motivation and application. Section 2 sets up the system model. In Section 3, we provide methods to construct a set of divergence packing centers (message set) and bounds for packing numbers. In Section 4, we present our new achievability bound and particularize this bound to the typical DMCs. Section 5 studies the asymptotic behavior of the achievability bound using Gaussian approximation analysis and applies it to the typical DMCs. In Section 6, we present numerical results. We conclude this paper in Section 7.

### 1.1. Motivation and Application

The noisy permutation channel models the scenario where codewords undergo reordering, which occurs in communication networks and DNA storage systems. We briefly outline some applications of this channel.

(a) *Communication Networks:* First, noisy permutation channels are a suitable model for the multipath routed network in which packets arrive at different delays [12,13]. In such networks, data packets within the same group often take paths of differing lengths, bandwidths, and congestion levels as they traverse the network to the receiver. Consequently, transmission delays exhibit unpredictable variations, causing these packets to arrive at their destination in a potentially different order from their original sending sequence. Moreover, during transmission, data packets may be lost or corrupted due to reasons such as link failures or buffer overflow. Treating all possible packets as the input alphabet, this scenario fits the noisy permutation channel model.
(b) *DNA Storage Systems:* The DNA storage systems, known for their high density and reliability over long periods, are another motivation for our research [14,15,16]. Such a system can be seen as an out-of-order communication channel [1,14,17]. The source data is written onto DNA molecules (or codeword strings) consisting of letters from an alphabet of four nucleotides {A,C,G,T}. Due to physical conditions causing random fragmentation of DNA molecules, long-read sequencing technology, such as nanopore sequencing [18], is employed at the receiver to read entire randomly permuted DNA molecules. In the noisy permutation channel, the DMC matrix models potential errors during the synthesis and storage of DNA molecules, followed by a random permutation block that represents the random permutation of DNA molecules. For a comprehensive overview of DNA storage systems, see [1,14]; studies presenting specific DNA-based storage coding schemes include [17,19,20].

### 1.2. Notation

We use [n]={1,…,n}, $Z_{\geq a}=\left\{a,a+1,a+2,\ldots \right\}$ to represent integer intervals. Let 1{·} denote the indicator function. For a given X and a random variable X∈X, we write $X∼P_{X}$ to indicate that the random variable X follows the distribution $P_{X}$. Let $X^{n}=\left(X_{1},\ldots ,X_{n}\right)$ and $x^{n}=\left(x_{1},\ldots ,x_{n}\right)$ denote the random vector and its realization in the *n*-th Cartesian product $X^{n}$, respectively. A (|X|−1)-dimensional simplex on $R^{\left|X\right|}$ is a set of points as follows:

(2) $$\Delta_{|X|-1}=\left\{\left(p_{1},p_{2},\ldots ,p_{\left|X\right|}\right)\in R^{\left|X\right|}|\;\sum_{x=1}^{\left|X\right|}p_{x}=1,p_{x}\geq 0\right\}.$$

The KL divergence and the total variation are denoted by D(·∥·) and TV(·,·), respectively. For a matrix *A*, we use the notation rank(A) to represent the rank of matrix *A*. The probability and mathematical expectation are denoted by P[·] and E[·], respectively. The cumulative distribution function of the standard normal distribution is denoted by Φ(·) and $\Phi^{-1}\left(\cdot \right)$ is its inverse function.

## 2. System Model

The code $C_{n}$ consists of a message set M, an (possibly randomized) encoder function $f_{n}:M\to X^{n}$, and a (possibly randomized) decoder function $g_{n}:Y^{n}\to M\cup \left\{e\right\}$, where the notation ‘e’ indicates that the decoder chooses “error”. We write X for the finite input alphabet and Y for the finite output alphabet. *M* denotes the achievable code size of code $C_{n}$.

The input alphabet X abstracts transmitted codeword symbols in various applications. For instance, in DNA storage applications, X denotes the alphabet of four nucleotides, while the *n*-length codeword $X^{n}\in X^{n}$ represents the DNA molecule formed by the corresponding *n* nucleotides. The sender uses encoder $f_{n}$ to encode the message *M* into a codeword $X^{n}$, which is then passed through the DMC *W* to produce $Z^{n}\in Y^{n}$. The DMC is defined by a |X|×|Y| matrix *W*, where W(z|x) denotes the probability that the output z∈Y occurs given input x∈X. Finally, $Z^{n}$ passes through a random permutation block $P_{Y^{n}|Z^{n}}$ to generate $Y^{n}\in Y^{n}$. The random permutation block $P_{Y^{n}|Z^{n}}$ operates as follows. First, a random permutation is denoted as σ:{1,…,n}↦{1,…,n}, drawn randomly and uniformly from the symmetric group $S_{n}$ over {1,…,n}. Then, $Y^{n}$ is generated by permuting $Z^{n}$ according to $Y_{i}=Z_{\sigma (i)}$ for all i∈{1,…,n}. The receiver uses decoder $g_{n}$ to produce the estimate of the message $\hat{M}$. We can describe these steps by the following Markov chain:

(3) $$M\to X^{n}\to Z^{n}\to Y^{n}\to \hat{M}.$$

**Figure 1:**
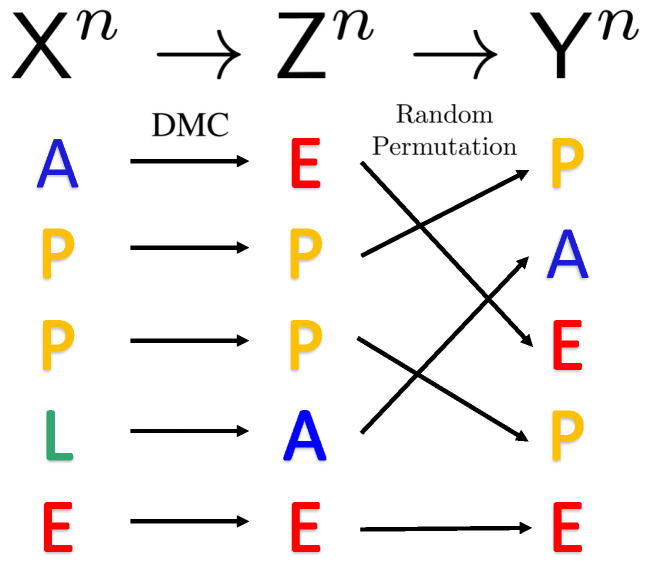
Illustration of a communication model with a DMC followed by a random permutation.

The channel model of the noisy permutation channel is illustrated in Figure 1. For codewords $X^{n}$ drawn i.i.d. from $P_{X}$, the random permutation block does not change the probability distribution of codewords [1], i.e., if $X^{n}\overset{i.i.d.}{∼}P_{X}$, then $Z^{n}\overset{i.i.d.}{∼}P_{X}$ and $Y^{n}\overset{i.i.d.}{∼}P_{X}$.

We say *W* is strictly positive if all the transition probabilities are greater than 0. We impose the following restrictions on the channel.

**Assumption** **1.** *The channel W is a strictly positive and full-rank square matrix.*

For a given code $C_{n}$, the average error probability of code $C_{n}$ is

(4) $$\begin{array}{c} P_{e}=P\left[M\neq \hat{M}\right]. \end{array}$$

The achievable code size with a given blocklength and probability of error is denoted by $M^{*}\left(n,\epsilon \right)=\max\left\{M\;|\;\exists \;C_{n}\;s.t.\;P_{e}\leq \epsilon \right\}.$ The code rate of the encoder–decoder pair $\left(f_{n},g_{n}\right)$ is denoted by

(5) $$R=\frac{\log M}{\log n},$$

where log(·) is the binary logarithm (with base 2) in this paper. Note that the rate R(n,ϵ) for the noisy permutation channel is not the conventional definition where $R\left(n\right)=\frac{1}{n}\log M$ since the noisy permutation channels will have rate 0 under this definition. The capacity for noisy permutation channels is defined as C=sup{R≥0:Risachievable}.

## 3. Message Set and Divergence Packing

A divergence packing is a set of centers in simplex $\Delta_{|Y|-1}$ such that the minimum distance between each center is greater than some KL distance. The following definition abides by the packing number.

**Definition** **1.** The achievability space of the marginal distribution is defined by $\Delta_{|Y|-1}^{*}=\left\{P_{X}\circ W\;|\;P_{X}\in \Delta_{|X|-1}\right\}$.

**Definition** **2.** Let $\left\{P_{1},\ldots ,P_{M}\right\}\subset \Delta_{|Y|-1}^{*}$ be the set of divergence packing centers. The divergence packing number on $\Delta_{|Y|-1}^{*}$ is defined by

(6) $$\begin{array}{c} N^{*}\left(r,|Y|\right)=\max\left\{M\;|\;\exists \left\{P_{1},\ldots ,P_{M}\right\}\;s.t.\;\min_{i\neq j}D\left(P_{i}∥P_{j}\right)\geq r\right\}, \end{array}$$

*where r>0 is the packing radius.*

Here, we provide some intuition for using divergence packing in noisy permutation channels. Using the ML decoder, we relate the non-asymptotic channel performance to the likelihood ratio of two distributions (decoding metric). The statistical mean of this decoding metric is the KL divergence, which arises from applying the law of large numbers as the blocklength grows. Thus, using divergence packing can obtain an upper bound of the error probability by lower bounding the distance between each distribution, and the code size can be analyzed asymptotically via the Berry–Esseen bound (see Section 5). These factors motivate us to use the KL divergence in constructing the message set.

Since the messages correspond to different distributions in noisy permutation channels, the message set can be equivalent to the set of marginal distributions at the receiver (e.g., see [1]). In the sequel, we denote the marginal distribution corresponding to message *m* by $P_{m}$.

Additionally, in Gaussian approximations, we need the following definition.

**Definition** **3.** Let $\mathrm{vol}(y,\Delta_{|Y|-1}^{*})$ and $\mathrm{vol}(y,\Delta_{|Y|-1})$ be, respectively, the volume of the projection of $\Delta_{|Y|-1}^{*}$ and $\Delta_{|Y|-1}$ from $R^{\left|Y\right|}$ to space $R^{|Y|-1}$, in which the y-th dimension is removed. The channel volume ratio is defined as

(7) $$V\left(W\right)=\max_{y\in Y}\frac{\mathrm{vol}(y,\Delta_{|Y|-1}^{*})}{\mathrm{vol}(y,\Delta_{|Y|-1})}.$$

Next, we present several lower bounds on packing numbers. In the two-dimensional case, our construction achieves tighter bounds. For higher dimensions, our primary tool is the volume bound for ϵ-packing (e.g., see [8] Theorem 27.3). These results form the foundation for constructing marginal probability distributions in subsequent sections, while also playing a key role in the analysis of Gaussian approximations.

### 3.1. Binary Case

We first give the lower bound of the packing number in the binary case. Consider $\Delta_{1}^{*}=\left\{\left(q,1-q\right)\;|\;\delta_{1}\leq q\leq 1-\delta_{2}\right\}$, where $0<\delta_{1}<1-\delta_{2}<1$. Define

(8) $$\begin{array}{c} \Gamma_{b,2}^{*}=\left\{\left(q,1-q\right)\;|\;q=\frac{\xi a}{\left\lfloor 1/b\right\rfloor }+\delta_{1}\;\mathrm{where}\;a\in Z_{\geq 0}\right\}, \end{array}$$

where $\xi =1-\delta_{1}-\delta_{2},b>0$.

Then, we have the following result proved in Appendix A.

**Proposition** **1.** Fix $\Delta_{1}^{*}=\left\{\left(q,1-q\right)\;|\;\delta_{1}\leq q\leq 1-\delta_{2}\right\}$, where $\delta_{1}>0$ and $\delta_{2}>0$. We can construct a set of packing centers by (8) with $b=\frac{1}{\xi }\sqrt{\frac{r}{2\log e}}$ and $\xi =1-\delta_{1}-\delta_{2}$ such that

(9) $$N^{*}\left(r,2\right)=\left\lfloor 1/r\right\rfloor +1.$$

### 3.2. General Case

Next, we introduce a general method for constructing the set of packing centers and the bounds of their size. Let b>0, and we consider the following set:

(10) $$\begin{array}{c} \Gamma_{b,|Y|}^{*}=\left\{P\in \Delta_{|Y|-1}^{*}|\begin{array}{c} P=\left(\frac{a_{1}}{\left\lfloor 1/b\right\rfloor },\ldots ,\frac{a_{\left|Y\right|}}{\left\lfloor 1/b\right\rfloor }\right)\;\mathrm{where}\;a_{1},\ldots ,a_{\left|Y\right|}\in Z_{\geq 0} \end{array}\right\}. \end{array}$$

The intuition behind constructing this set is that the minimum distance of distributions in this uniform structure can be bounded by the total variation distance. Then, we can obtain the set of divergence packing centers that have a certain radius by applying Pinsker’s inequality.

We have the following lower bound proved in Appendix A.

**Theorem** **1.** Fix a W that satisfies Assumption 1 and generate $\Delta_{|Y|-1}^{*}$. We can construct a set of packing centers by (10) with $b=\sqrt{\frac{r}{2\log e}}$ such that

(11) $$\begin{array}{c} N^{*}\left(r,|Y|\right)\geq V\left(W\right){\left(\frac{\log e}{8r}\right)}^{\frac{|Y|-1}{2}}, \end{array}$$

*where r>0 is the packing radius.*

## 4. New Bounds on Rate

In this section, we introduce our new bound, which is based on divergence packing and yields the spirit of the RCU bound. The key ingredient is our analysis of error events.

To that end, we introduce some definitions. Suppose we have a set of marginal distributions M, which is constructed by (8) or (10) with any b>0. Fixing a $P=(p_{1},\ldots ,p_{\left|Y\right|})\in M$, we are often concerned with the divergence packing centers close to *P*. To do this, we consider $Q_{a,b}\left(P\right)=\left(q_{1},\ldots ,q_{\left|Y\right|}\right)$, where a≠b, $q_{a}=p_{a}+\frac{K}{\left\lfloor 1/r\right\rfloor },q_{b}=p_{b}-\frac{K}{\left\lfloor 1/r\right\rfloor }$ and $q_{i}=p_{i}$ for i∈Y∖{a,b}. *K* is a constant that has value ξ or 1 when M is constructed by (8) or (10), respectively. We define $R_{P}$ as the neighboring set of *P*:

(12) $$R_{P}=\left\{Q_{a,b}\left(P\right)|\;a,b\in Y,a\neq b\right\}\cap M.$$

In general, the distribution $Q_{a,b}\left(P\right)$ coincides with distributions in the set M, except near the boundaries of the simplex where $Q_{a,b}\left(P\right)$ may violate the constraints of the probability space. We use the intersection operation in (12) to make sure all elements of $R_{P}$ remain within the simplex. For convenience, we use $j\in [|R_{P}|]$ to index $Q_{j}\in R_{P}$, and we say $Q_{j}\in R_{P}$ is the neighboring distribution of *P*. By counting, we have |RP|≤2(|Y|2).

For the marginal distribution $P_{m}$ corresponding to the transmitted message *m*, we use the log-likelihood ratio to define the following decoding metric:

(13) $$d\left(m,j,y\right):=\log\frac{P_{m}\left(y\right)}{Q_{j}\left(y\right)},$$

where $Q_{j}\in R_{P_{m}}$.

Then, the proof of our main result consists of three parts, each detailed in one of the following subsections. In the first subsection, we introduce a lemma. This lemma shows that the message sets constructed by (8) or (10) have an overlapping relationship for error events. In the second subsection, we use this lemma to give an equivalent expression for the error probability. The third subsection contains our main result. Additionally, we particularize this new bound to BSC and BEC permutation channels in the fourth and fifth subsections, respectively.

### 4.1. Overlapping of Error Events

Intuitively, the rate of decay of $P_{e}$ is dominated by the rate of decay of the probability of error in distinguishing neighboring messages. In order to use this intuition mathematically, we need to analyze the relationship between error events. The following lemma, proved in Appendix B, does this and can be used for analyzing random coding bounds.

**Lemma** **1.** *Let M be constructed by (8) or (10) with any b>0. Fix a P∈M. Then, for every $\Lambda =\left(\lambda_{1},\ldots ,\lambda_{\left|Y\right|}\right)\in \Delta_{|Y|-1}$ and $Q=\left(q_{1},\ldots ,q_{\left|Y\right|}\right)\in M\setminus \left(R_{P}\cap \left\{P\right\}\right)$, if*

(14) $$\prod_{i=1}^{\left|Y\right|}p_{i}^{\lambda_{i}}\leq \prod_{i=1}^{\left|Y\right|}q_{i}^{\lambda_{i}},$$

there exists a $Q^{*}=\left(q_{1}^{*},\ldots ,q_{\left|Y\right|}^{*}\right)\in R_{P}$ such that

(15) $$\prod_{i=1}^{\left|Y\right|}p_{i}^{\lambda_{i}}\leq \prod_{i=1}^{\left|Y\right|}{\left(q_{i}^{*}\right)}^{\lambda_{i}}.$$

### 4.2. Equivalent Expression

In this subsection, we give a lemma tailored to our purposes. It follows directly from Lemma 1.

**Lemma** **2.** *For the set of marginal distributions M constructed by (8) or (10) with any b>0, we have*

(16) $$\begin{array}{c} P\left[\overset{\left|M\right|}{\underset{j=1,j\neq m}{⋃}}\left\{P_{j}^{n}\left(Y^{n}\right)\geq P_{m}^{n}\left(Y^{n}\right)\right\}\right]=P\left[\overset{|R_{P_{m}}|}{\underset{j=1}{⋃}}\left\{Q_{j}^{n}\left(Y^{n}\right)\geq P_{m}^{n}\left(Y^{n}\right)\right\}\right], \end{array}$$

*where the sequence $Y^{n}$ is drawn i.i.d. from $P_{m}$ and $Q_{j}\in R_{P_{m}}$.*

**Proof.**  Using Lemma 1, for j∈[|M|], if $\left\{P_{j}^{n}\left(y^{n}\right)\geq P_{m}^{n}\left(y^{n}\right)\right\}$ occurs, we obtain $j\in [|R_{P_{m}}|]$ such that $\left\{Q_{j}^{n}\left(y^{n}\right)\geq P_{m}^{n}\left(y^{n}\right)\right\}$ occurs. Then, we observe

(17) $$P\left[\overset{\left|M\right|}{\underset{j=1,j\neq m}{⋃}}\left\{P_{j}^{n}\left(Y^{n}\right)\geq P_{m}^{n}\left(Y^{n}\right)\right\}\right]=\sum_{y^{n}\in Y^{n}}P_{m}^{n}\left(y^{n}\right)\overset{\left|M\right|}{\underset{j=1,j\neq m}{⋃}}1\left\{P_{j}^{n}\left(y^{n}\right)\geq P_{m}^{n}\left(y^{n}\right)\right\}$$

(18) $$=\sum_{y^{n}\in Y^{n}}P_{m}^{n}\left(y^{n}\right)\overset{|R_{P_{m}}|}{\underset{j=1}{⋃}}1\left\{Q_{j}^{n}\left(y^{n}\right)\geq P_{m}^{n}\left(y^{n}\right)\right\}.$$

(19) $$=P\left[\overset{|R_{P_{m}}|}{\underset{j=1}{⋃}}\left\{Q_{j}^{n}\left(Y^{n}\right)\geq P_{m}^{n}\left(Y^{n}\right)\right\}\right],$$

where in (17) we sum over all possible outputs, and (18) relies on Lemma 1 by setting Λ to be the empirical distribution of $y^{n}$. This completes the proof of (16). □

**Remark** **1.** *If the transmitted message is m, Lemma 2 shows that the union of error events $\cup_{j=1,j\neq m}^{\left|M\right|}\left\{P_{j}^{n}\geq P_{m}^{n}\right\}$ can be equivalent to a minor union $\cup_{j=1}^{|R_{P_{m}}|}\left\{Q_{j}^{n}\geq P_{m}^{n}\right\}$ on $R_{P_{m}}$. Its size depends on the size of the output alphabet.*

### 4.3. Main Result: New Lower Bound

The main result in this section is the following. Please refer to Appendix C for the proof.

**Theorem** **2.** Fix a W that satisfies Assumption 1 and generate $\Delta_{|Y|-1}^{*}$. Let the set of marginal distributions M be constructed by (8) or (10) with any b>0. Then, there exists a code $C_{n}$ (average error probability) with achievable code size |M| such that

(20) $$\begin{array}{c} \epsilon \leq \min\left\{1,\;\frac{1}{\left|M\right|}\sum_{m=1}^{\left|M\right|}\sum_{j=1}^{|R_{P_{m}}|}\sum_{y^{n}\in Y^{n}}P_{m}^{n}\left(y^{n}\right)\cdot 1\left\{\sum_{i=1}^{n}d\left(m,j,y_{i}\right)\leq 0\right\}\right\}. \end{array}$$

**Remark** **2.** Theorem 2 relies on the message set constructed by (8) or (10). We restrict the channel W to be a full-rank square matrix, which makes $\Delta_{|Y|-1}^{*}$ an equal-dimensional subspace of $\Delta_{|Y|-1}$. Therefore, the evenly spaced grid structure on $\Delta_{|Y|-1}^{*}$ can be constructed by using (8) or (10). Without this condition, we cannot apply (10) unless we make strong assumptions about W.

**Remark** **3.** *Theorem 2 upper bounds the probability of error with the sum of the probabilities of error events on $R_{P_{m}}$ instead of M, which makes our bound much stronger than [1]. In fact, if we do not use Lemma 2 but instead apply the union bound and the second moment method for TV distance ([21] Lemma 4.2(iii)) in the proof of Theorem 2, we can obtain the existing bound ([1] Equation (36)).*

### 4.4. BSC Permutation Channels

In this subsection, we particularize the nonasymptotic bounds to the BSC, i.e., the DMC matrix is

(21) $$W=\left[\begin{array}{cc} 1-\delta & \delta \\ \delta & 1-\delta \end{array}\right],$$

denoted ${\mathrm{BSC}}_{\delta }$. According to Proposition 1 and Theorem 1, using (8) to construct the set of marginal distributions is better than (10) in the binary case. Therefore, we focus on the former in this subsection. For convenience, we denote $P_{m}\left(\cdot \right)=\left(\delta_{m},1-\delta_{m}\right)$. For i,j∈[|M|], let $\delta_{i}<\delta_{j}$ if i<j. Then, for a $P_{m}$, we clearly have

(22) $$R_{P_{m}}=\left\{\begin{array}{cc} \{P_{m-1},P_{m+1}\}, & 2\leq m\leq |M|-1,\\ \{P_{2}\}, & m=1,\\ \{P_{|M|-1}\}, & m=|M|. \end{array}\right.$$

Let

(23) f1(n,Ti)=∑t=0Tintδit(1−δi)n−t,i≥2,0,i=1,

and

(24) f2(n,Ti)=∑t=Tinntδit(1−δi)n−t,i≤|M|−1,0,i=|M|.

The following bound is a straightforward generalization of Theorem 2.

**Theorem** **3** (Achievability)**.** *For the BSC permutation channel with crossover probability δ, there exists a code $C_{n}$ such that*

(25) $$\begin{array}{c} \epsilon \leq \frac{\sum_{i=1}^{\left|M\right|}}{\left|M\right|}\min\left\{1,f_{1}\left(n,{\underline{T}}_{i}\right)+f_{2}\left(n,{\bar{T}}_{i}\right)\right\}, \end{array}$$

*where*

(26) $${\underline{T}}_{i}=\left\lfloor \frac{n\log\frac{1-\delta_{i-1}}{1-\delta_{i}}}{\log\frac{\delta_{i}\left(1-\delta_{i-1}\right)}{\delta_{i-1}\left(1-\delta_{i}\right)}}\right\rfloor$$

*and*

(27) $${\bar{T}}_{i}=\left\lceil \frac{n\log\frac{1-\delta_{i+1}}{1-\delta_{i}}}{\log\frac{\delta_{i}\left(1-\delta_{i+1}\right)}{\delta_{i+1}\left(1-\delta_{i}\right)}}\right\rceil .$$

*The set of marginal distributions is constructed by (8) and for the radius r, we have*

(28) |M|=⌊1/r⌋+1.

**Proof.**  Let us assume the transmitted message is m∈M, corresponding to the marginal distribution $P_{m}$. In BSC, we focus on the set (22). Using the same argument in the proof of Lemma 1, the term corresponding to $d(m,m-1,y_{i})$ in (20) can be computed as

(29) ∑yn∈YnPmn(yn)·1∑i=1nd(m,m−1,yi)≤0=∑t=0T¯mntδmt(1−δm)n−t,

where ${\underline{T}}_{m}$ follows from (A19). Similarly, the term corresponding to $d(m,m+1,y_{i})$ in (20) can be computed as

(30) ∑yn∈YnPmn(yn)·1∑i=1nd(m,m+1,yi)≤0=∑t=T¯mnntδmt(1−δm)n−t,

where ${\bar{T}}_{m}$ follows from (A19). Equations (29) and (30) are substituted into Theorem 2 to complete the proof. □

### 4.5. BEC Permutation Channels

The BEC permutation channel with erasure probability δ consists of input alphabet X={0,1} and output alphabet Y={0,e,1}, where the conditional distribution is

(31) $$\forall z\in Y,\forall x\in X,W\left(z|x\right)=\left\{\begin{array}{cc} 1-\delta , & z=x,\\ \delta , & z=e,\\ 0, & \mathrm{otherwise}. \end{array}\right.$$

Moreover, we denote such a channel as ${\mathrm{BEC}}_{\delta }$ for convenience.

Next, we have the following achievability bound.

**Proposition** **2.** *For BEC permutation channels with erasure probability 2δ, there exists a code $C_{n}$ such that the average probability of error and the code size satisfy (25) and (28), respectively.*

**Proof.**  The derivations of this proof follow from ([1] Proposition 6), and we include the details for the sake of completeness. We first note that the BSC matrix satisfies the Doeblin minorization condition (e.g., see [1] Definition 5) with $1^{⊤}/2$ and constant 2δ. Using ([1] Lemma 6), we find that ${\mathrm{BSC}}_{\delta }$ is a degraded version of ${\mathrm{BEC}}_{2\delta }$. Then, for the encoder and decoder pairs $\left(f_{n},g_{n}\right)$ for BSC permutation channels and $\left(f_{n},{\tilde{g}}_{n}\right)$ for BEC permutation channels, the average probability of error satisfies [1] Equation (36).

(32) $$P_{e}\left(f_{n},g_{n},{\mathrm{BSC}}_{\delta }\right)=P_{e}\left(f_{n},{\tilde{g}}_{n},{\mathrm{BEC}}_{2\delta }\right).$$

Then, the argument of the proof of Theorem 3 is repeated. This completes the proof. □

## 5. Gaussian Approximation

We turn to the asymptotic analysis of the noisy permutation channel for a given blocklength and average probability of error.

### 5.1. Auxiliary Lemmata

To establish our Gaussian approximation, we will present two lemmata. The first lemma we will exploit is an important tool in the Gaussian approximation analysis:

**Lemma** **3** (*Berry–Esseen, [22] Chapter XVI.5, Theorem 2*)**.** *Fix a positive integer n. Let $Z_{i}$ indexed by (1,…,n) be independent. Then, for any real x and $C_{0}\leq 6$ we have*

(33) $$\left|P\left[\sum_{i=1}^{n}Z_{i}<n\left(\mu_{n}+x\sqrt{\frac{V_{n}}{n}}\right)\right]-\Phi \left(x\right)\right|\leq \frac{B_{n}}{\sqrt{n}},$$

*where*

(34) $$\begin{array}{c} \mu_{n}=\frac{1}{n}\sum_{i=1}^{n}E\left[Z_{i}\right],\;V_{n}=\frac{1}{n}\sum_{i=1}^{n}\mathrm{Var}\left[Z_{i}\right], \end{array}$$

(35) $$\begin{array}{c} T_{n}=\frac{1}{n}\sum_{i=1}^{n}E\left[{\left|Z_{i}-\mu_{i}\right|}^{3}\right],\;B_{n}=C_{0}\frac{T_{n}}{V_{n}^{3/2}}. \end{array}$$

To develop the Gaussian approximation, we consider the following definitions. The variance and third absolute moment of log-likelihood ratio between two distributions *P* and *Q* are defined as $V\left(P∥Q\right)=E\left[{\left(\log\frac{P}{Q}-D\left(P∥Q\right)\right)}^{2}\right]$ and $T\left(P∥Q\right)=E\left[{\left|\log\frac{P}{Q}-D\left(P∥Q\right)\right|}^{3}\right]$, respectively. Then, the following lemma is concerned with properties of V(P∥Q) and T(P∥Q), which is proved in Appendix D.

**Lemma** **4.** Fix a W that satisfies Assumption 1 and generate $\Delta_{|Y|-1}^{*}$. Let M constructed by (10) with any b>0 be the set of packing centers on $\Delta_{|Y|-1}^{*}$. If packing radius $r\leq \frac{2\log e}{9}$, for any P∈M and $Q\in R_{P}$, we have

(36) $$V\left(P∥Q\right)=rF_{0},$$

*where*

(37) $$\left(\frac{5}{8p_{max}}-\frac{2}{9}\right)\log e\leq F_{0}\leq \frac{5\log e}{2p_{min}{\left(1-p_{max}\right)}^{2}},$$

*$p_{min}$ and $p_{max}$ are constants greater than 0. Additionally, we have*

(38) $$T\left(P∥Q\right)\leq \frac{36\sqrt{2}{\left(\log e\right)}^{3/2}}{p_{min}^{2}{\left(1-p_{max}\right)}^{3}}r_{0}^{3/2}.$$

### 5.2. Main Result: Gaussian Approximation

The main result in this section is the following. Please refer to Appendix E for the proof.

**Theorem** **4.** *Fix W is a strictly positive and full-rank square matrix for noisy permutation channels. Then, there exists a number $N_{0}\geq 1$, such that*

(39) $$\log M^{*}\left(n,\epsilon \right)=\ell \log\left(\frac{\sqrt{n}}{-\Phi^{-1}\left(\epsilon /G\right)}\right)+\log V\left(W\right)+\theta$$

*is achievable for all $n\geq N_{0}$, where ℓ=rank(W)−1, G=2ℓ+12, V(W) is the channel volume ratio, and θ is a constant.*

This achievable code size (39) is different from the Gaussian approximation of the traditional channel (e.g., see [7]) since our bound is obtained by divergence packing numbers $N^{*}\left(r,|Y|\right)$. The packing radius is a key ingredient affecting the lower bound of $N^{*}\left(r,|Y|\right)$ and affects the error probability.

### 5.3. Approximation of BSC and BEC Permutation Channels

We apply Theorem 4 to obtain the following approximation.

**Corollary** **1.** *For BSC permutation channels with crossover probability δ, there exists a number $N_{0}\geq 1$, such that*

(40) $$\log M^{*}\left(n,\epsilon \right)=\log\left(\frac{\left(1-2\delta \right)\sqrt{n}}{-\Phi^{-1}\left(\frac{\epsilon }{2}\right)}\right)+\theta$$

*is achievable for all $n\geq N_{0}$, where θ is a constant.*

**Proof.**  For BSC, we have $\Delta_{1}^{*}=\left\{\left(p,1-p\right)\;|\;\delta \leq p\leq 1-\delta \right\}$. By using Lagrange’s formula [23], we have $V({\mathrm{BSC}}_{\delta })=1-2\delta$. Substituting this into Theorem 4 yields the result. □

**Remark** **4.** *We remark that the Gaussian approximation shows some properties of the code size with a given blocklength n and probability of error ϵ. In BSC permutation channels, while the channel capacity is only related to the rank of the channel matrix, the rate at which the achievable code size approaches the capacity is affected by crossover probability δ.*

The approximation of BSC permutation channels can also be derived from Proposition 1. To use the message set constructed by (8), we need the following lemma, which is proved in Appendix D:

**Lemma** **5.** Fix a W that satisfies Assumption 1 and generate $\Delta_{1}^{*}$. Let M constructed by (8) with any b>0 be the set of packing centers on $\Delta_{1}^{*}$. Then, there exists a packing radius $r_{1}$, such that for all $r\leq r_{1}$, we have

(41) $$F_{0}r\leq V\left(P∥Q\right)\leq F_{1}r$$

*and*

(42) $$T\left(P∥Q\right)\leq F_{2}r^{3/2},$$

*where $F_{0}$, $F_{1}$ and $F_{2}$ are positive and finite.*

Then, we have the following result.

**Proposition** **3.** *For BSC permutation channels with crossover probability δ, there exists a number $N_{0}\geq 1$, such that*

(43) $$\log M^{*}\left(n,\epsilon \right)=\log\left(\left\lceil \frac{\left(1-2\delta \right)\sqrt{n}}{-\Phi^{-1}\left(\frac{\epsilon }{2}\right)}\right\rceil \right)+\theta$$

*is achievable for all $n\geq N_{0}$, where θ is a constant.*

**Proof.**  Instead of using (10), we use (8) with $\Delta_{1}^{*}=\left\{\left(p,1-p\right)\;|\;\delta \leq p\leq 1-\delta \right\}$. Repeat the argument of the proof of Theorem 4 replacing Lemma 4 with Lemma 5. Note that for M constructed by (8) with any b>0, we have

(44) $$b=\frac{1}{1-2\delta }\sqrt{\frac{r}{2\log e}}.$$

We use Proposition 1 to continue as follows:

(45) $$\begin{array}{cc} \log M^{*}\left(n,\epsilon \right) & \geq \log\left(\left\lfloor \frac{\left(1-2\delta \right)\sqrt{n}}{-\Phi^{-1}\left(\frac{\epsilon }{2}\right)}\right\rfloor +1\right)+\log F_{0}\\ \; & \geq \log\left(\left\lceil \frac{\left(1-2\delta \right)\sqrt{n}}{-\Phi^{-1}\left(\frac{\epsilon }{2}\right)}\right\rceil \right)+\log F_{0}, \end{array}$$

where $F_{0}\geq 0$ is a constant. This completes the proof. □

Next, for BEC permutation channels, we have the following approximation.

**Proposition** **4.** *For BEC permutation channels with erasure probability η, there exists a number $N_{0}\geq 1$, such that*

(46) $$\log M^{*}\left(n,\epsilon \right)=\log\left(\frac{\left(1-\eta \right)\sqrt{n}}{-\Phi^{-1}\left(\frac{\epsilon }{2}\right)}\right)+\theta$$

*and*

(47) $$\log M^{*}\left(n,\epsilon \right)=\log\left(\left\lceil \frac{\left(1-\eta \right)\sqrt{n}}{-\Phi^{-1}\left(\frac{\epsilon }{2}\right)}\right\rceil \right)+\theta$$

*are achievable for all $n\geq N_{0}$, where θ is a constant.*

**Proof.**  Through Theorem 2, repeat the argument of the proof of Corollary 1 and Theorem 3, replacing δ with η/2. □

## 6. Numerical Results

In this section, we perform numerical evaluations to illustrate our results. We first validate the precision of our Gaussian approximation across a wide range of parameters. Secondly, we present the performance of bounds of a binary DNA storage system and compare them with existing bounds.

### 6.1. Precision of the Gaussian Approximation

**Figure 2:**
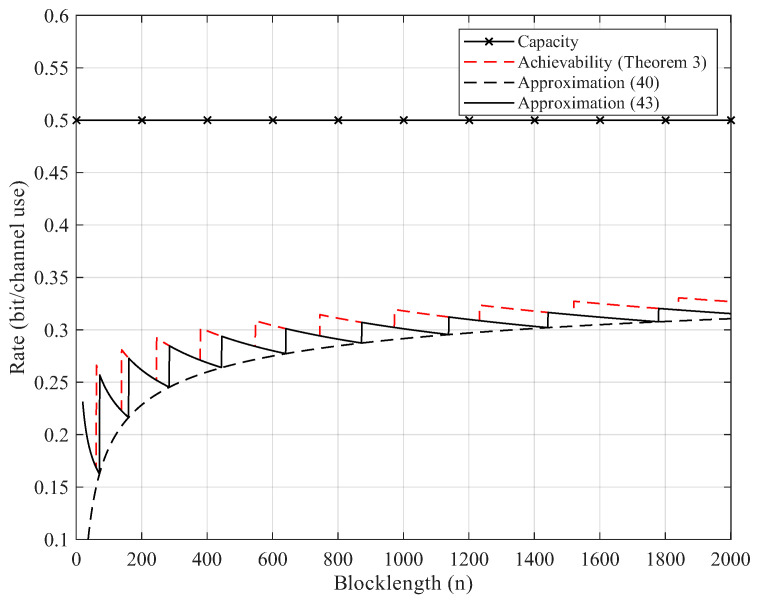
Rate–blocklength tradeoff for the BSC with crossover probability δ=0.11 and average block error rate $\epsilon =10^{-3}$.

**Figure 3:**
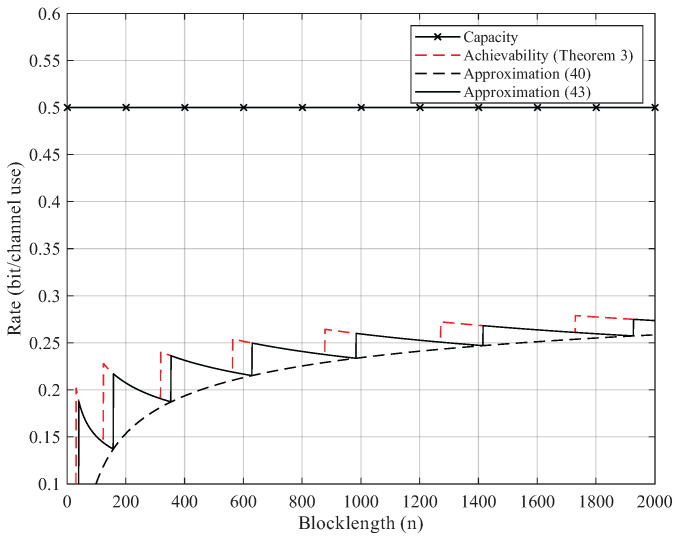
Rate–blocklength tradeoff for the BSC with crossover probability δ=0.11 and average block error rate $\epsilon =10^{-6}$.

**Figure 4:**
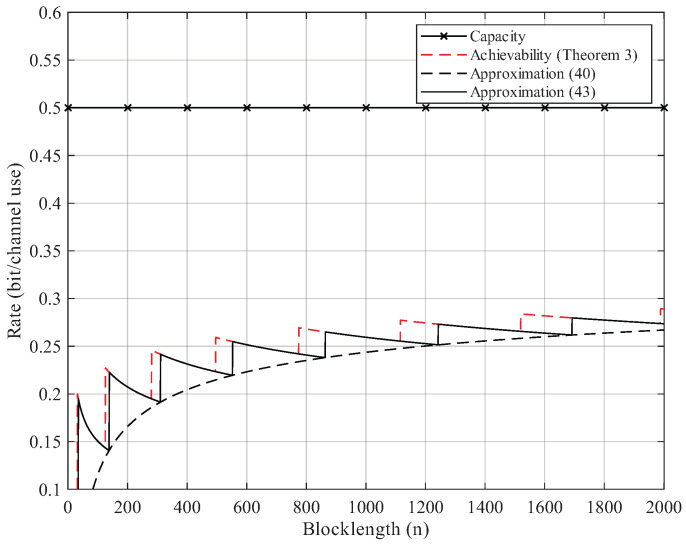
Rate–blocklength tradeoff for the BSC with crossover probability δ=0.22 and average block error rate $\epsilon =10^{-3}$.

**Figure 5:**
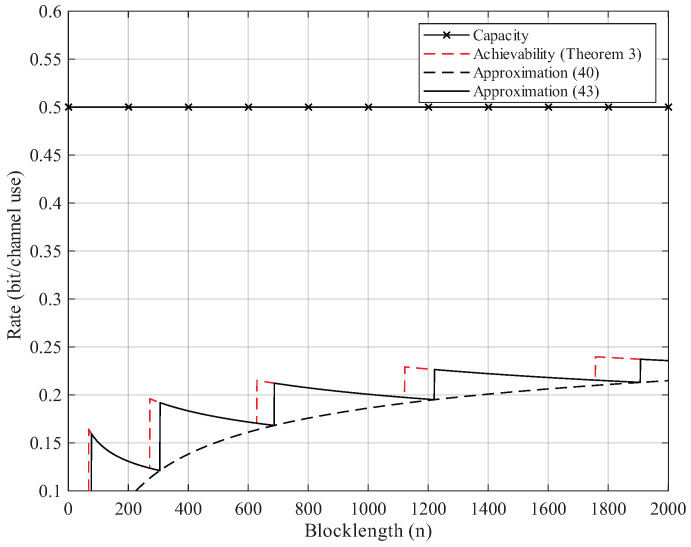
Rate–blocklength tradeoff for the BSC with crossover probability δ=0.22 and average block error rate $\epsilon =10^{-6}$.

Here, we give the numerical results. According to Proposition 2, the BEC permutation channel with erasure probability 2δ can be equivalent to the BSC permutation channel with crossover probability δ. Thus, we focus on the numerical results of BSC permutation channels. We use Theorem 3 to compute the non-asymptotic achievability bound. We start searching from M=2 until the right side of (25) is less than the error probability ϵ. For Gaussian approximation, we use (40) and (43) but omit the remainder term θ. As Figure 2, Figure 3, Figure 4 and Figure 5 show, although the remainder term of the Gaussian approximation is a constant, it is still quite close to the non-asymptotic achievability bound. In fact, for all n≥20, the difference between (43) and Theorem 3 is within 1 bit in $\log M^{*}\left(n,\epsilon \right)$.

### 6.2. Comparison with Existing Bound

**Figure 6:**
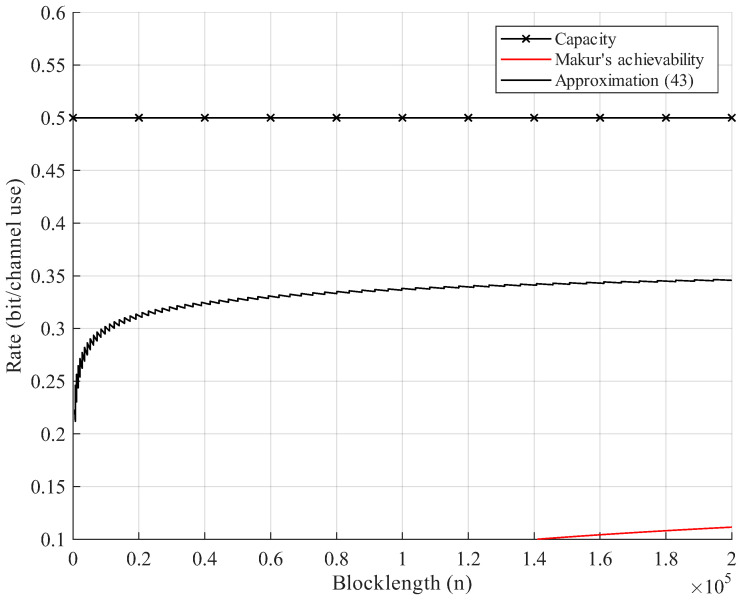
Rate–blocklength tradeoff for the BSC with crossover probability δ=0.25 and average block error rate $\epsilon =10^{-3}$: example of a DNA storage system.

Additionally, in the context of DNA storage systems, we consider codewords composed of nucleotides {A,C,G,T}. For simplicity, *A* and *C* are regarded as the symbol 0, and *G* and *T* are regarded as the symbol 1 in code constructions, giving a binary alphabet {0,1}. The synthesis errors and random permutation of DNA molecules are modeled as the BSC Permutation Channel with crossover probability δ=0.25. To reduce the computation complexity, we use approximation (43). Furthermore, we present numerical results for the existing lower bound, namely, Makur’s achievability bound ([1] Equation (36)) for BSC permutation channels. The result shows that our new achievability bound is uniformly better than Makur’s bound. In the setup of Figure 6, our bound quickly approaches half of the capacity (n≈1000). As the blocklength increases, Makur’s bound reaches 20% of the channel capacity at about $n\approx 1.4\times 10^{5}$, shown in Figure 6. This is because we show the overlapping relationship of error events, which reduces the number of error events when applying the union bound.

## 7. Conclusions and Discussion

In summary, we established a new achievability bound for noisy permutation channels with a strictly positive and full-rank square matrix. The key element is that our analysis indicates that the size of error events in the union is independent of the message set. This allows us to derive a refined asymptotic analysis of the achievable rate. Numerical simulations show that our new achievability bound is stronger than Makur’s achievability bound in [1]. Additionally, our approximation is quite accurate, even though the remainder term is a constant. Finally, the primary future work will generalize the DMC matrix in noisy permutation channels to non-full-rank and non-strictly positive matrices. Other future work may improve asymptotic expansion (e.g., improving the remainder term to o(1)).

## Data Availability

The original contributions presented in this study are included in the article. Further inquiries can be directed to the corresponding author.

## Appendix A. Divergence Packing

### Appendix A.1. Proof of Proposition 1

We note that $\Gamma_{b,2}^{*}$ constructed by (8) is a subset of $\Delta_{1}^{*}$. Fix $b=\frac{1}{\xi }\sqrt{\frac{r}{2\log e}}$. Then, for $P,Q\in \Gamma_{b,2}$ we have

(A1) $$\min_{P\neq Q}\left(2\log e\right){\mathrm{TV}}^{2}\left(P,Q\right)\geq r.$$

Use Pinsker’s inequality ([8] Theorem 7.10) to obtain $\min_{P\neq Q}D\left(P∥Q\right)\geq r$. Then, we conclude this proof by realizing that $|\Gamma_{b,2}^{*}|=\left\lfloor 1/b\right\rfloor +1$.

### Appendix A.2. Proof of Theorem 1

To prove Theorem 1, we consider the set

(A2) $$\begin{array}{c} \Gamma_{b,|Y|}=\left\{P\in \Delta_{|Y|-1}\;|\;\begin{array}{c} P=\left(\frac{a_{1}}{\left\lfloor 1/b\right\rfloor },\ldots ,\frac{a_{\left|Y\right|}}{\left\lfloor 1/b\right\rfloor }\right)\;\mathrm{where}\;a_{1},\ldots ,a_{\left|Y\right|}\in Z_{\geq 0} \end{array}\right\}. \end{array}$$

We have the following lemma:

**Lemma** **A1.**
*Let $\Gamma_{b,|Y|}$ be constructed by (A2) with any b>0. For $P,Q\in \Gamma_{b,|Y|}$, we have*

(A3)
$$\min_{P\neq Q}\mathrm{TV}\left(P,Q\right)\geq b.$$

**Proof.** For every $P\in \Gamma_{b,|Y|}$, we can find a $Q\in \Gamma_{b,|Y|}$ such that for m,n∈Y and m≠n, we have $q_{m}\neq p_{m}$, $q_{n}\neq p_{n}$. For i∈Y∖{m,n}, we have $q_{i}=p_{i}$. Then, we obtain that $\min_{P\neq Q}\mathrm{TV}\left(P,Q\right)=\min_{P\neq Q}\frac{1}{2}\sum_{i\in Y}\left|p_{i}-q_{i}\right|=\frac{1}{\left\lfloor 1/b\right\rfloor }\geq b$. □

Fix a radius $b=\sqrt{\frac{r}{2\log e}}>0$ and fix a $\Gamma_{b,|Y|}^{*}$ constructed by (10). Note that we have $\Gamma_{b,|Y|}^{*}\subset \Gamma_{b,|Y|}$. For any $P,Q\in \Gamma_{b,|Y|}^{*}$, we have

(A4) $$\min_{P\neq Q}D\left(P∥Q\right)\overset{\left(a\right)}{\geq }\min_{P\neq Q}\left(2\log e\right){\mathrm{TV}}^{2}\left(P,Q\right)\overset{\left(b\right)}{\geq }r,$$

where (a) follows from Pinsker’s inequality and (b) follows from Lemma A1. On the other hand, note that the right inequality of (A4) shows that

(A5) $$\min\sum_{y\in Y}\left|P\left(y\right)-Q\left(y\right)\right|\geq \sqrt{\frac{2}{\log e}}\sqrt{r}.$$

Hence, a total variation packing constructed by $\Gamma_{b,|Y|}^{*}$ can be regarded as an $\ell_{1}$-norm packing of $\Delta_{|Y|-1}^{*}$ with radius $\sqrt{\frac{2r}{\log e}}$. Let $B_{\ell_{1}}^{|Y|-1}$ be the $\ell_{1}$-norm unit ball. The volume bound ([8] Theorem 27.3) of $\ell_{1}$-norm packing provides the lower bound of $|\Gamma_{b,|Y|}^{*}|$ as

(A6) $$\left|\Gamma_{b,|Y|}^{*}\right|\geq {\left(\frac{\log e}{2r}\right)}^{\frac{|Y|-1}{2}}\frac{\mathrm{vol}(y,\Delta_{|Y|-1}^{*})}{\mathrm{vol}(B_{\ell_{1}}^{|Y|-1})}.$$

Here, $\mathrm{vol}(B_{\ell_{1}}^{|Y|-1})$ is the volume of the $\ell_{1}$-norm unit ball; $\mathrm{vol}(y,\Delta_{|Y|-1}^{*})$ is the volume of the projection of $\Delta_{|Y|-1}^{*}$ from $R^{\left|Y\right|}$ to the space $R^{|Y|-1}$, consistent with the argument used in the proof of ([24] Proposition 2).

Note that the maximum volume ratio is V(W) by Definition 3. We continue the bounding as follows:

(A7) $$\begin{array}{cc} |\Gamma_{b,|Y|}^{*}|\; & \geq {\left(\frac{\log e}{2r}\right)}^{\frac{|Y|-1}{2}}\times \frac{V\left(W\right)\mathrm{vol}\left(y,\Delta_{|Y|-1}\right)}{\mathrm{vol}(B_{\ell_{1}}^{|Y|-1})}, \end{array}$$

(A8) $$\begin{array}{cc} \; & =V\left(W\right){\left(\frac{\log e}{8r}\right)}^{\frac{|Y|-1}{2}}, \end{array}$$

where

- (A7) holds since this projection can remove any *y*-th dimension, where y∈Y. Consequently, the lower bound is given by taking the maximum of the volume ratio over y∈Y;
- (A8) follows by using Lagrange’s formula [23] and the volume formula to obtain $\mathrm{vol}\left(y,\Delta_{|Y|-1}^{*}\right)=\frac{V(W)}{(|Y|-1)!}$ and $\mathrm{vol}\left(B_{\ell_{1}}^{|Y|-1}\right)=\frac{2^{|Y|-1}}{(|Y|-1)!}$, respectively.

Finally, we conclude the proof by realizing that the left inequality of (A4) implies that $\Gamma_{b,|Y|}^{*}$ gives the set of divergence packing centers. This completes the proof of Theorem 1.

## Appendix B. Proof of Lemma 1

We first consider M constructed by (10). Fix a $P=(p_{1},\ldots ,p_{\left|Y\right|})\in M$ and generate $R_{P}$ corresponding to *P*. Fix a,b∈Y, where a≠b. Denote a $Q_{a,b}^{*}=\left(q_{1}^{*},\ldots ,q_{\left|Y\right|}^{*}\right)$ such that $q_{a}^{*}=p_{a}+\frac{1}{\left\lfloor 1/r\right\rfloor }$, $q_{b}^{*}=p_{b}-\frac{1}{\left\lfloor 1/r\right\rfloor }$, $q_{i}^{*}=p_{i}$, where i∈Y∖{a,b}. Let

(A9) $$\left|\log\frac{p_{a}}{p_{b}+1/\left\lfloor 1/r\right\rfloor }\right|=G_{a,b}\left|\log\frac{p_{b}}{p_{b}-1/\left\lfloor 1/r\right\rfloor }\right|,$$

where $G_{a,b}$ is a constant. Define

(A10) $$B_{a,b}=\left\{\left(\lambda_{1},\ldots ,\lambda_{\left|Y\right|}\right)\;|\;G_{a,b}\lambda_{a}<\lambda_{b}\right\}.$$

If $Q_{a,b}^{*}\in R_{P}$, for $\Lambda =\left(\lambda_{1},\ldots ,\lambda_{\left|Y\right|}\right)\in B_{a,b}$ and $Q_{a,b}^{*}=\left(q_{1}^{*},\ldots ,q_{\left|Y\right|}^{*}\right)$, we have

(A11) $$\prod_{i=1}^{\left|Y\right|}p_{i}^{\lambda_{i}}>\prod_{i=1}^{\left|Y\right|}{\left(q_{i}^{*}\right)}^{\lambda_{i}}.$$

For a,b∈Y and a≠b, we find $B_{a,b}$ and consider the intersection, i.e., $B_{P}={⋂}_{a,b\in Y,a\neq b,Q_{a,b}^{*}\in R_{P}}B_{a,b}$. Then, for $\Lambda \in B_{P}$, we have (A11) holds for any $Q^{*}\in R_{P}$.

Now we consider $Q\in M\setminus (R_{P}\cap \left\{P\right\}$ ), which is farther from *P* in Euclidean distance than $Q^{*}\in R_{P}$. For each i∈Y, we obtain that the Euclidean distance between $p_{i}$ and $q_{i}$ is $\frac{K_{i}}{\left\lfloor 1/r\right\rfloor }$, where $K_{i}\in Z_{\geq 0}$.

Since *Q* is a probability distribution, we have $p_{i}-\frac{K_{i}}{\left\lfloor 1/r\right\rfloor }>0$ and $K_{i}\geq 0$. Using the inequality ${\left(1+p_{i}\right)}^{K}\geq 1+Kp_{i}$, we obtain that

(A12) $$K_{i}\left|\log\frac{p_{i}}{p_{i}+1/\left\lfloor 1/r\right\rfloor }\right|\geq \left|\log\frac{p_{i}}{p_{i}+K_{i}/\left\lfloor 1/r\right\rfloor }\right|$$

and

(A13) $$K_{i}\left|\log\frac{p_{i}}{p_{i}-1/\left\lfloor 1/r\right\rfloor }\right|\leq \left|\log\frac{p_{i}}{p_{i}-K_{i}/\left\lfloor 1/r\right\rfloor }\right|.$$

Let $Y^{*}=\left\{i\in Y\;|\;q_{i}\neq p_{i}\right\}$, $Y_{0}=\left\{i\in Y^{*}\;|\;q_{i}<p_{i}\right\}$, and $Y_{1}=\left\{i\in Y^{*}\;|\;q_{i}>p_{i}\right\}$. For $i\in Y_{0}$, let $q_{i}^{\prime }=p_{i}-1/\left\lfloor 1/r\right\rfloor$. For $i\in Y_{1}$, let $q_{i}^{\prime }=p_{i}+1/\left\lfloor 1/r\right\rfloor$. Then, we have

(A14) $$\sum_{i\in Y^{*}}\lambda_{i}\log\frac{p_{i}}{q_{i}}\geq \sum_{i\in Y^{*}}\lambda_{i}K_{i}\log\frac{p_{i}}{q_{i}^{\prime }}.$$

Due to the constraints of the probability space, we have

(A15) $$\sum_{i\in Y_{0}}K_{i}=\sum_{i\in Y_{1}}K_{i}.$$

Recall that for $\Lambda \in B_{P}$, (A11) holds for any $Q^{*}\in R_{P}$. Then, by the definition of $R_{P}$, for $\Lambda \in B_{P}$, we have

(A16) $$\lambda_{a}\log\frac{p_{a}}{q_{a}^{\prime }}+\lambda_{b}\log\frac{p_{b}}{q_{b}^{\prime }}>0,$$

where $a\in Y_{0}$ and $b\in Y_{1}$. We combine (A14)–(A16) to obtain that if $\Lambda \in B_{P}$, then

(A17) $$\sum_{i\in Y^{*}}\lambda_{i}\log\frac{p_{i}}{q_{i}}>0.$$

That is

(A18) $$\prod_{i=1}^{\left|Y\right|}p_{i}^{\lambda_{i}}>\prod_{i=1}^{\left|Y\right|}q_{i}^{\lambda_{i}}.$$

For $Q\in M\setminus (R_{P}\cap \left\{P\right\})$, define $B_{Q}$ such that (A17) holds for $\Lambda \in B_{Q}$. We have $B_{P}\subset B_{Q}$. Denote by $A_{Q}=\Delta_{|Y|-1}\setminus B_{Q}$ and $A_{P}=\Delta_{|Y|-1}\setminus B_{P}$ the complement of $B_{Q}$ and $B_{P}$, respectively. We note that if (14) holds, we have $\Lambda \in A_{Q}$. Then, we obtain (15) holds for a $Q^{*}\in R_{P}$ since we obviously have $A_{Q}\subset A_{P}$. This completes the proof in the case of M constructed by (10).

We then give another proof in binary case. Consider M constructed by (8). For convenience, let $p_{j}<p_{m}$ if j<m, where j,m∈[|M|]. Fix $P_{m-1}=\left(p_{m-1},1-p_{m-1}\right)$ and $P_{m}=\left(p_{m},1-p_{m}\right)$. Let

(A19) $$f\left(p_{j}\right)=\left(\log\frac{1-p_{j}}{1-p_{m}}\right)/\left(\log\frac{p_{m}\left(1-p_{j}\right)}{p_{j}\left(1-p_{m}\right)}\right).$$

For any λ∈[0,1] and j∈[m−2], we have

(A20) $$p_{j}^{\lambda }{\left(1-p_{j}\right)}^{\left(1-\lambda \right)}\geq p_{m}^{\lambda }{\left(1-p_{m}\right)}^{\left(1-\lambda \right)}$$

holds for $\lambda \in [0,f\left(p_{j}\right)]$. Note that $f(p_{j})$ is a monotonically increasing function with respect to $p_{j}$. Then, we can obtain

(A21) $$p_{m-1}^{\lambda }{\left(1-p_{m-1}\right)}^{\left(1-\lambda \right)}\geq p_{m}^{\lambda }{\left(1-p_{m}\right)}^{\left(1-\lambda \right)}$$

since $\left[0,f\left(p_{j}\right)\right]\subset \left[0,f\left(p_{m-1}\right)\right]$.

For j∈{m+1,m+3,…,|M|}, use the same argument to obtain that (A20) holds for $\lambda \in [f\left(p_{j}\right),1]$ and

(A22) $$p_{m+1}^{\lambda }{\left(1-p_{m+1}\right)}^{\left(1-\lambda \right)}\geq p_{m}^{\lambda }{\left(1-p_{m}\right)}^{\left(1-\lambda \right)}$$

holds for $\lambda \in [f\left(p_{m+1}\right),1]$. Then, (A20) implies (A22) since $\left[f\left(p_{j}\right),1\right]\subset \left[f\left(p_{m-1}\right),1\right]$. This completes the proof.

## Appendix C. Proof of Theorem 2

Since the matrix is full-rank, we obtain the achievability space of the marginal distribution is a (|Y|−1)-dimensional probability space $\Delta_{|Y|-1}^{*}$. We consider constructing the set of marginal distributions M by using the divergence packing on space $\Delta_{|Y|-1}^{*}$, i.e., let M be constructed by (8) or (10) with any b>0.

Assuming the transmitted message is *m*, corresponding to the marginal distribution $P_{m}$. After the codeword $X^{n}$ passes through the DMC matrix *W* and the random permutation block $P_{Y^{n}|Z^{n}}$, we obtain that $Y^{n}\overset{i.i.d.}{∼}P_{m}$. Then, error event of the maximum likelihood decoder is

(A23) $$\overset{\left|M\right|}{\underset{j=1,j\neq m}{⋃}}\left\{P_{j}^{n}\left(Y^{n}\right)\geq P_{m}^{n}\left(Y^{n}\right)\right\}.$$

Then, for $Y^{n}$ drawn i.i.d. from $P_{m}$ the average probability of error satisfies

(A24) $$P[\mathrm{error}|M=m]\leq P\left[\overset{\left|M\right|}{\underset{j=1,j\neq m}{⋃}}\left\{P_{j}^{n}\left(Y^{n}\right)\geq P_{m}^{n}\left(Y^{n}\right)\right\}\right]$$

(A25) $$=P\left[\overset{|R_{P_{m}}|}{\underset{j=1}{⋃}}\left\{P_{j}^{n}\left(Y^{n}\right)\geq P_{m}^{n}\left(Y^{n}\right)\right\}\right]$$

(A26) $$\leq \sum_{j=1}^{|R_{P_{m}}|}P\left[\left\{P_{j}^{n}\left(Y^{n}\right)\geq P_{m}^{n}\left(Y^{n}\right)\right\}\right],$$

where

- (A24) follows from we regard the equality case, $P_{j}^{n}\left(Y^{n}\right)\geq P_{1}^{n}\left(Y^{n}\right)$, as an error event though the ML decoder might return the correct message;
- (A25) follows from Lemma 2;
- (A26) follows from the union bound.

Let the message be uniform on M. Then, we take the expectation over all codewords to obtain

(A27) $$\epsilon \leq \frac{1}{\left|M\right|}\sum_{m=1}^{\left|M\right|}\sum_{j=1}^{|R_{P_{m}}|}\sum_{y^{n}\in Y^{n}}P_{m}^{n}\left(y^{n}\right)1\left\{\sum_{i=1}^{n}d\left(m,j,y_{i}\right)\leq 0\right\}$$

This completes the proof.

## Appendix D. Properties of V(P∥Q) and T(P∥Q)

This appendix is concerned with the behavior of V(P∥Q) and T(P∥Q). We first prove Lemma 4.

### Appendix D.1. Proof of Lemma 4

Since the DMC matrix is strictly positive, each term of marginal distribution *P* is uniformly bounded away from zero, i.e., there exists a $p_{\min}\in \left(0,1\right)$ such that $P\left(y\right)\geq p_{\min}$ for all y∈Y. Similarly, there exists a $p_{\max}\in \left(0,1\right)$ such that $P\left(y\right)\leq p_{\max}$ for all y∈Y. Clearly, we have $p_{\min}=\min_{y\in Y}\left\{P\left(y\right)\;|\;P\in M\right\}$ and $p_{\max}=\max_{y\in Y}\left\{P\left(y\right)\;|\;P\in \Delta_{|Y|-1}^{*}\right\}$. Without loss of generality, we assume |M|≥2. That is, we have $r_{0}\leq \frac{2\log e}{9}$.

We know that

(A28) $$\begin{array}{cc} P(y) & =\frac{a}{1/r-\delta }\\ \; & =\frac{a\sqrt{\frac{r_{0}}{2\log e}}}{1-\delta \sqrt{\frac{r_{0}}{2\log e}}}, \end{array}$$

where δ∈[0,1) and $a\in Z_{\geq 1}$.

We consider each term P(y) in *P* separately. If P(y)=Q(y), we obviously get zero. If P(y)>Q(y), we have $Q\left(y\right)=\frac{\left(a-1\right)\sqrt{\frac{r_{0}}{2\log e}}}{1-\delta \sqrt{\frac{r_{0}}{2\log e}}}$. Here, since the distributions in M have no zero term, we have $a\in Z_{\geq 2}$. Since $a=P\left(y\right)\left(1-\delta \sqrt{\frac{r_{0}}{2\log e}}\right)\sqrt{\frac{2\log e}{r_{0}}}$, we have

(A29) $$\begin{array}{c} \frac{\sqrt{r_{0}\log e}}{P\left(y\right)\left(\sqrt{2}-\delta \sqrt{r_{0}/\log e}\right)}\leq \log\frac{P(y)}{Q(y)}\leq \frac{\sqrt{r_{0}\log e}}{P\left(y\right)\left(\sqrt{2}-\delta \sqrt{r_{0}/\log e}\right)-\sqrt{r_{0}/\log e}}. \end{array}$$

Consequently, we have

(A30) $$P\left(y\right){\left(\log\frac{P(y)}{Q(y)}\right)}^{2}\geq \frac{r_{0}\log e}{2P(y)}$$

and

(A31) $$\begin{array}{c} P\left(y\right){\left(\log\frac{P(y)}{Q(y)}\right)}^{2}\leq \frac{r_{0}\log e}{2{\left(a-1\right)}^{2}P\left(y\right)/a^{2}}\leq \frac{2r_{0}\log e}{P(y)}{\left(\frac{1}{1-\delta \sqrt{\frac{r_{0}}{2\log e}}}\right)}^{2}. \end{array}$$

If P(y)<Q(y), consider $Q\left(y\right)=\frac{\left(a+1\right)\sqrt{\frac{r_{0}}{2\log e}}}{1-\delta \sqrt{\frac{r_{0}}{2\log e}}}$, where $a\in Z_{\geq 1}$. Applying the same argument to obtain

(A32) $$\begin{array}{c} \frac{\sqrt{r_{0}\log e}}{P\left(y\right)\left(\sqrt{2}-\delta \sqrt{r_{0}/\log e}\right)+\sqrt{r_{0}/\log e}}\leq |\log\frac{P(y)}{Q(y)}|\leq \frac{\sqrt{r_{0}\log e}}{P\left(y\right)\left(\sqrt{2}-\delta \sqrt{r_{0}/\log e}\right)}. \end{array}$$

Consequently, we have

(A33) $$\begin{array}{c} P\left(y\right){\left(\log\frac{P(y)}{Q(y)}\right)}^{2}\geq \frac{r_{0}\log e}{2{\left(a+1\right)}^{2}P\left(y\right)/a^{2}}\geq \frac{r_{0}\log e}{8P(y)} \end{array}$$

and

(A34) $$\begin{array}{cc} P\left(y\right){\left(\log\frac{P(y)}{Q(y)}\right)}^{2}\; & \leq \frac{r_{0}\log e}{2P(y)}{\left(\frac{1}{1-\delta \sqrt{\frac{r_{0}}{2\log e}}}\right)}^{2}. \end{array}$$

Then, we see that

(A35) $$V(P∥Q)\geq -D{\left(P∥Q\right)}^{2}+\sum_{y}P\left(y\right){\left(\log\frac{P(y)}{Q(y)}\right)}^{2}$$

(A36) $$\geq r_{0}\log e\left(\frac{5}{8p_{max}}-\frac{r_{0}}{\log e}\right),$$

where

- (A35) simply expand the variance;
- (A36) holds since the definition of $R_{P}$ ensures that for all y∈Y, we only have two terms that are not zero and can be bounded by (A30) and (A33), respectively.

Finally, we complete the proof of the lower bound by noting that $r_{0}\leq \frac{2\log e}{9}$.

Note that

(A37) $${\left(\frac{1}{1-\delta \sqrt{\frac{r_{0}}{2\log e}}}\right)}^{2}\leq \frac{1}{{\left(1-p_{max}\right)}^{2}}.$$

Using (A31) and (A34), we obtain the upper bound

(A38) $$V(P∥Q)\leq \frac{5\log e}{2p_{min}{\left(1-p_{max}\right)}^{2}}r_{0},$$

which yields (36).

We now turn to bound T(P∥Q). For P(y)>Q(y), we have

(A39) $$P\left(y\right)|\log\frac{P(y)}{Q(y)}{|}^{3}\leq \frac{4}{\sqrt{2}}\frac{{\left(r_{0}\log e\right)}^{3/2}}{P^{2}\left(y\right)}{\left(\frac{1}{1-\delta \sqrt{\frac{r_{0}}{2\log e}}}\right)}^{3}.$$

For P(y)<Q(y), we have

(A40) $$P\left(y\right)|\log\frac{P(y)}{Q(y)}{|}^{3}\leq \frac{1}{2\sqrt{2}}\frac{{\left(r_{0}\log e\right)}^{3/2}}{P^{2}\left(y\right)}{\left(\frac{1}{1-\delta \sqrt{\frac{r_{0}}{2\log e}}}\right)}^{3}.$$

Note that D(P∥Q) can be bounded by the following:

(A41) $$D\left(P∥Q\right)\leq \frac{3\sqrt{2\log e}}{2}\sqrt{r_{0}}\left(\frac{1}{1-\delta \sqrt{\frac{r_{0}}{2\log e}}}\right).$$

Using the inequality ${\left|a-b\right|}^{3}\leq {4(|a|}^{3}+{\left|b\right|}^{3})$, we obtain

(A42) $$T(P∥Q)\leq \frac{36\sqrt{2}{\left(\log e\right)}^{3/2}}{p_{min}^{2}{\left(1-p_{max}\right)}^{3}}r_{0}^{3/2},$$

which establishes (38).

### Appendix D.2. Proof of Lemma 5

Then, we prove Lemma 5 for M constructed by (8). We first note that

(A43) $$\begin{array}{cc} P(y) & =\frac{a\xi }{1/r-\delta }\\ \; & =\frac{\left(a-\frac{\delta_{1}\delta }{\xi }+F_{0}\right)\sqrt{\frac{r_{0}}{2\log e}}}{1-\frac{\delta }{\xi }\sqrt{\frac{r_{0}}{2\log e}}}, \end{array}$$

where δ∈[0,1], $F_{0}=\delta_{1}/\sqrt{\frac{r_{0}}{2\log e}}$, and $a\in Z_{\geq 1}$. Note that $F_{0}-\frac{\delta_{1}\delta }{\xi }\geq 0$ holds for small $r_{0}$. Repeat the proof of Lemma 4, replacing *a* with $a-\frac{\delta_{1}\delta }{\xi }+F_{0}$ and δ with $\frac{\delta }{\xi }$. Then, there exists a packing radius $r_{1}$, such that for all $r_{0}\leq r_{1}$, we have

(A44) $$F_{0}r_{0}\leq V\left(P∥Q\right)\leq F_{1}r_{0}$$

and

(A45) $$T\left(P∥Q\right)\leq F_{2}r_{0}^{3/2},$$

where $F_{0}$, $F_{1}$ and $F_{2}$ are positive and finite.

## Appendix E. Proof of Theorem 4

Let $M_{r}$ constructed by (10) with $b=\sqrt{\frac{r}{2\log e}}$ be the set of packing centers on $\Delta_{|Y|-1}^{*}$, where r>0 is the packing radius and we specify later. For the transmitted message *m*, passing codeword through the DMC matrix $W^{n}$ and random permutation block $P_{Y^{n}|Z^{n}}$ induces a marginal distribution $P_{m}$ on $Y^{n}$. Note that the decoding metric is the sum of independent identically distributed variables:

(A46) $$\sum_{k=1}^{n}d\left(m,j,Y_{k}\right)=\sum_{k=1}^{n}\log\frac{P_{m}\left(Y_{k}\right)}{Q_{j}\left(Y_{k}\right)}.$$

It has the mean $D(P_{m}∥Q_{j})$, variance $V(P_{m}∥Q_{j})$, and third absolute moment $T(P_{m}∥Q_{j})$. Denote

(A47) $$B_{m}=\max_{j\in [|R_{P_{m}}|]}\frac{6T(P_{m}∥Q_{j})}{\sqrt{V^{3}\left(P_{m}∥Q_{j}\right)}}$$

and

(A48) $$P_{e}\left[m\right]=\sum_{j=1}^{|R_{P_{m}}|}P\left[\sum_{k=1}^{n}d\left(m,j,Y_{k}\right)\leq 0\right].$$

According to Theorem 2, there exists a code with average error probability $\epsilon^{\prime }$ such that

(A49) $$\epsilon^{\prime }\leq \frac{1}{|M_{r}|}\sum_{m=1}^{|M_{r}|}P_{e}\left[m\right].$$

Denote $m^{*}=\underset{m\in M}{\mathrm{argmax}}P_{e}\left[m\right]$. We continue (A49) as follows:

(A50) $$\epsilon^{\prime }\leq P_{e}\left[m^{*}\right]$$

(A51) $$\leq \frac{|R_{P_{m^{*}}}|B_{m^{*}}}{\sqrt{n}}+\max_{j\in [|R_{P_{m^{*}}}|]}\left|R_{P_{m^{*}}}\right|\Phi \left(\frac{-nD(P_{m^{*}}∥Q_{j})}{\sqrt{nV(P_{m^{*}}∥Q_{j})}}\right)$$

(A52) $$\leq \frac{|R_{P_{m^{*}}}|B_{m^{*}}}{\sqrt{n}}+\left|R_{P_{m^{*}}}\right|\Phi \left(-\sqrt{\frac{nr_{0}}{F_{0}}}\right),$$

where

- (A51) follows from Theorem 3;
- (A52) holds for a suitable constant $F_{0}>0$ by Lemma 4.

Equating the RHS of (A52) to ϵ and noting that $\epsilon \geq \epsilon^{\prime }>0$, we solve that

(A53) $$r_{0}=\frac{{\left(\Phi^{-1}\left(\frac{\epsilon }{|R_{P_{m^{*}}}|}-\frac{F_{1}}{\sqrt{n}}\right)\right)}^{2}F_{0}}{n},$$

where |RPm*|≤2(|Y|2) by its definition, $F_{0}>0$ and $F_{1}>0$ are suitable constants. This can be done since for suitable constants $F_{2}>0$ and $F_{3}>0$, we obtain $T\left(P_{m}∥Q_{j}\right)\leq F_{2}r_{0}^{3/2}$ and $V\left(P_{m}∥Q_{j}\right)\geq F_{3}r_{0}$ for *n* sufficiently large by Lemma 4. Consequently, for large *n*, $B_{m}$ can be upper bounded by a suitable constant $F_{1}$.

The above arguments indicate that there exists a code with average error probability ϵ such that the code size $|M_{r}|$ is achievable. Let ℓ=|Y|−1 and let G=2(|Y|2). For *n* sufficiently large, we obtain that for suitable $F_{5}>0$,

(A54) $$\log\left|M_{r}\right|=\log N^{*}\left(r,|Y|\right)$$

(A55) $$\geq \log\left(V\left(W\right){\left(\frac{\log e}{8}\right)}^{\frac{\ell }{2}}{\left(\frac{\sqrt{n}F_{4}}{-\Phi^{-1}\left(\epsilon /G\right)}\right)}^{\ell }\right)$$

(A56) $$\geq \ell \log\left(\frac{\sqrt{n}}{-\Phi^{-1}\left(\epsilon /G\right)}\right)+\log V\left(W\right)+\log F_{5},$$

where

- (A54) follows from that $M_{r}$ constructed by (10) is the set of packing centers and has the packing number $N^{*}\left(r,|Y|\right)$;
- (A55) holds for suitable $F_{4}>0$ by Theorem 1 and Taylor’s formula of $\Phi^{-1}\left(\cdot \right)$.

This completes the proof.

## References

[1] Makur A. (2020). Coding Theorems for Noisy Permutation Channels. IEEE Trans. Inf. Theory.

[2] Makur A. Bounds on Permutation Channel Capacity. Proceedings of the 2020 IEEE International Symposium on Information Theory (ISIT).

[3] Tang J., Polyanskiy Y. (2023). Capacity of Noisy Permutation Channels. IEEE Trans. Inf. Theory.

[4] Feng L., Wang B., Lv G., Li X., Wang L., Jin Y. (2025). New Upper Bounds for Noisy Permutation Channels. IEEE Trans. Commun..

[5] Strassen V. Asymptotic Estimates in Shannon’s Information Theory. Proceedings of the Transactions of the Third Prague Conference on Information Theory.

[6] Hayashi M. (2009). Information Spectrum Approach to Second-Order Coding Rate in Channel Coding. IEEE Trans. Inf. Theory.

[7] Polyanskiy Y., Poor H.V., Verdu S. (2010). Channel Coding Rate in the Finite Blocklength Regime. IEEE Trans. Inf. Theory.

[8] Polyanskiy Y., Yihong W. (2023). Information Theory: From Coding to Learning.

[9] Polyanskiy Y. (2010). Channel Coding: Non-Asymptotic Fundamental Limits. Ph.D. Thesis.

[10] Kolmogorov A.N. (1993). Selected Works of A. N. Kolmogorov. Mathematics and Its Applications.

[11] Yuhong Y., Andrew B. (1999). Information-theoretic determination of minimax rates of convergence. Ann. Stat..

[12] John W., Steven W., Wa M. Optimal rate delay tradeoffs for multipath routed and network coded networks. Proceedings of the 2008 IEEE International Symposium on Information Theory (ISIT).

[13] John W., Steven W., Wa M. (2009). Optimal Rate–Delay Tradeoffs and Delay Mitigating Codes for Multipath Routed and Network Coded Networks. IEEE Trans. Inf. Theory.

[14] Yazdi S.M., Hossein T., Han M., Garcia R. (2015). DNA-Based Storage: Trends and Methods. IEEE Trans. Mol. Biol. Multi-Scale Commun..

[15] Erlich T., Zielinski D. (2017). DNA Fountain enables a robust and efficient storage architecture. Science.

[16] Heckel R., Shomorony I., Ramchandran K., Tse D.N.C. Fundamental limits of DNA storage systems. Proceedings of the 2017 IEEE International Symposium on Information Theory (ISIT).

[17] Kovačević M., Tan V.Y. (2018). Codes in the Space of Multisets—Coding for Permutation Channels with Impairments. IEEE Trans. Inf. Theory.

[18] Laver T., Harrison J., Moore K., Farbos A., Paszkiewicz K., Studholme D. (2015). Assessing the performance of the oxford nanopore technologies minion. J. Mol. Biol..

[19] Kovačević M., Tan V.Y. (2018). Asymptotically optimal codes correcting fixed-length duplication errors in DNA storage systems. IEEE Commun. Lett..

[20] Kiah M.H., Puleo G., Milenkovic O. (2016). Codes for DNA sequence profiles. IEEE Trans. Inf. Theory.

[21] Evans W., Kenyon C., Peres Y., Schulman L.J. (2000). Broadcasting on trees and the Ising model. Ann. Appl. Prob..

[22] Feller W. (1971). An Introduction to Probability Theory and Its Applications.

[23] Stein P. (1966). A Note on the Volume of a Simplex. Am. Math. Mon..

[24] Jennifer T. (2021). Divergence Covering. Ph.D. Thesis.

